# The Influence of a Health-Related Fitness Training Program on Motor Performance as Well as Hematological and Biochemical Parameters

**DOI:** 10.3390/ijerph17020578

**Published:** 2020-01-16

**Authors:** Dorota Kostrzewa-Nowak, Anna Nowakowska, Teresa Zwierko, Maciej Rybak, Robert Nowak

**Affiliations:** 1 Centre for Human Structural and Functional Research, University of Szczecin, 17C Narutowicza St., 70-240 Szczecin, Poland; anna.nowakowska@usz.edu.pl (A.N.); teresa.zwierko@usz.edu.pl (T.Z.); robert.nowak@usz.edu.pl (R.N.); 2 Student of “Sports Diagnostics” and “Physical Education”, Faculty of Physical Education and Health, University of Szczecin, 70-240 Szczecin, Poland; maciazrybak@gmail.com

**Keywords:** blood, health exercise program, motor performance, physical activity

## Abstract

The study was aimed at designing a health exercise program appealing to inactive young men, and then testing the men’s metabolic responses to the program using common diagnostic markers of general health. Six men, aged 22–29 years, took a part in training program to increase their motor performance and improve general health conditions. Body composition parameters, clinical chemistry variables (metabolites, albumin, total protein, ferritin, C reactive protein, lipid profile, ions, and selected enzymes activities) and blood morphology parameters were determined. Motor performance measured before and after a 4-month-long macrocycle indicated an increase in endurance, pace, and agility of the participants. Significant differences were found in analyzed enzymes activities. There was a significant increase in C-reactive protein levels from pre- to post-training. Additionally, changes in hematological biomarkers were seen that suggest erythropoiesis might significantly increase, specifically during the last 2-month-long mesocycles. The proposed training program induced small improvements in endurance, pace, and agility. It was also confirmed that changes in aspartate (AST) and alanine (ALT) activities emerge before any increase in creatine kinase (CK) activity that is important in monitoring of the training loads. Observed changes in red blood cell-related parameters suggest increase in erythropoiesis in the second half of the training cycle.

## 1. Introduction

Physical activity (PA) is one of the significant factors positively influencing the body mass index (BMI), body composition, and cardiorespiratory fitness. Consequently, PA represents an important component of prevention and/or attenuation of chronic diseases, including diabetes mellitus, cancer, obesity, hypertension, osteoporosis and osteoarthritis, and depression [1]. The benefits of PA on psychosocial and cognitive health have been also confirmed [2,3,4]. Further, PA has a beneficial effect on general health conditions and induces metabolic changes that are manifested in hematological changes of clinical biochemistry parameters [5,6,7,8]. Health-Related Fitness (H-RF), which is developed during various training programs, is also associated with an improvement of the general well-being. Moreover, it is worth noting that people who undertake recreational training still require specific training programs to maintain health and acquire proper habits. The promotion of PA as a way of helping to maintain or improve health should minimize the risk of injury, which can occur after application of excessive load on the body. It seems that the implementation of an optimal short-term training plan can not only improve motor performance but also restore good H-RF habits [9,10,11,12].

A common feature of professional and recreational sports is providing a stress to the body, which enhances metabolic processes through various ways. As the goals of competitive and recreational training are quite different, it follows that recreational training is focused mainly on enhancing catabolic processes; whereas in competitive training, where catabolic processes are necessary only to produce energy, the training program is designed to stimulate anabolic processes that improve athletes’ performance. It is well known that energy turnover is the main factor influencing metabolism. Hence, regular PA induces metabolic changes and can alter the serum concentrations of numerous biomarkers. These modifications can often lead to measured outcomes outside the reference value ranges, leading to additional examinations for the athlete and/or discontinuation of training and competition [7,13]. Previous studies emphasize that because long-term training influences athletes’ cellular metabolism, muscle damage, and measures of oxidative stress [7,14,15], metabolic biomarkers in athletes’ blood can often be found at clinical diagnostic levels [15,16,17,18].

Experts at the World Health Organization (WHO) have published global recommendations on physical activity for health advising at least 30 min of moderate-intensity PA for five days a week. Studies have also identified that healthy PA levels can be determined based on average daily steps achieved by an individual, in particular for sedentary: <5000 steps per day, for active: 5000–9999 steps per day, and for highly active: ≥10,000 steps per day [19]. Taking all that into account, it is important to offer daily exercise programs with regard for age and physical fitness and experience. PA programs should support taking up activities appropriate for individual health and should be introduced gradually. The aim of this study was to create a training program targeted to physically inactive young men, who were physically active in the past and evaluate the effect of it on wide range of health-related parameters. During 4 months of intervention the individual status of physical adaptation on a cellular/molecular level was systematically measured.

## 2. Materials and Methods

### 2.1. Participants

Six former regional soccer players aged 22–29 years that were physically inactive (sedentary) during the last 2 years were included in this study. All participants who qualified for the study took part in the same macrocycle training program described below. All laboratory tests were conducted at the same time (of day and of the macrocycle) for each participant. Participants had no history of any metabolic syndrome (according to the International Diabetes Federation description) [20] or cardiovascular diseases (defined by WHO) [21]. They were non-smokers, non-drinkers, and refrained from taking any medications or supplements known to affect metabolism. The study was approved by the Local Ethics Committee in accordance with the Helsinki Declaration.

### 2.2. Training Program

**Figure 1:**
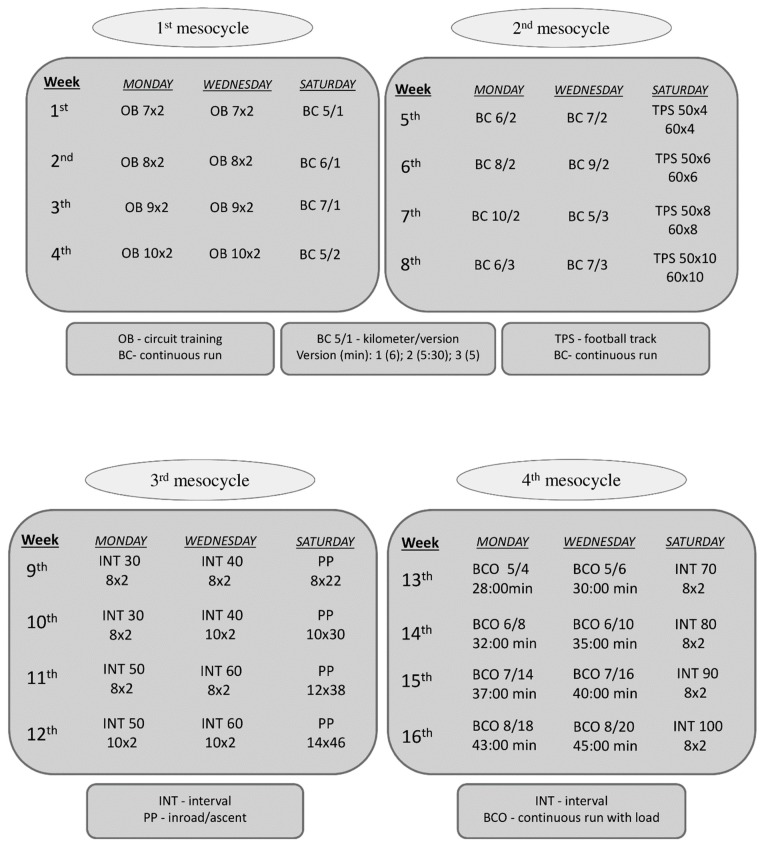
Macrocycle training protocol. OB 7 × 2—seven exercises × two circuits; TPS 50 × 4—length of football track (m) × number of repetitions, 60 × 4—length of “parachute run” (m) × number of lengths covered; INT 30 (8 × 2)—interval including 30 m of sprint (number of 330 m—long laps × 2 min per lap); PP 8 × 22—number of inroads × number of ascents; BCO 5/4—distance of run (km) × load (kg), below there is a time for completion of the BCO exercise.

The training program was focused on two goals. The first was to increase the motor performance, specifically endurance, pace, and agility, of the participants, and the second was to examine outcome measures of general health. The macrocycle including four different mesocycles and lasted from December to March (Figure 1).

Before and after the 4-month training cycle, tests to determine motor skills (i.e., endurance, pace, and agility) were performed. Each mesocycle was focused on improving a specific component of motor performance and was divided into four stages: (i) circuit training and continuous runs, (ii) football tracks and continuous runs, (iii) uphill and interval running, (iii) running intervals and continuous runs with a load.

In the warm-up (5 min) and cool-down (10 min) periods, stabilizing exercises, plyometric exercises, stretching, relaxing exercises, massage objects (rollers), and coordination exercises (sensorimotor balls) were used.

The circuit training (in the 1st mesocycle) was a regular station training including exercises such as rompers, skipping on a skipping rope, boxer, kickboxer, triangle, skier, bricklayer, eights, skip A, jumping hurdles on both feet, jumping through hurdles with one leg and an element of stabilization, envelope, football pentagon, defense attack (gallop sideways forward and backward), bermuda triangle, football slalom, football slalom and pass, vertical ladder (skip A), horizontal ladder (skip A), sideways jumps. During each week the time schedule of the whole exercise was as follows: 1st (two circuits, seven exercises each) 20 s-10 s-2 min; 2nd (two circuits, eight exercises each) 25 s-5 s-1 min; 3rd (two circuits, nine exercises each) 30 s-5 s-30 s; and 4th (two circuits, 10 exercises each) 30 s-0 s-20 s, for exercise–between exercise rest–between circuits rest, respectively.

The length of football track (in the 2nd mesocycle) was 50 m and the number of exercises and repetitions increased in each week of the mesocycle (Figure 1). The schedule of the track was as follows:

(5th week) Four exercises (slalom without ball, jumping on both legs, diagonal sprint (zigzag), fast skip A through the training ladder), two repetitions of the track (30 s for the return to the start point), 2 min rest and two more repetitions of the track (30 s for the return to the start point);

(6th week) Six exercises (slalom without ball, jumping on both legs, slalom without ball, diagonal sprint (zigzag), exit to play, fast skip A through the training ladder), two repetitions of the track (25 s for the return to the start point), 2 min rest, two repetitions of the track (30 s for the return to the start point), 2 min rest and two more repetitions of the track (30 s for the return to the start point);

(7th week) Six exercises (slalom without ball, jumping on both legs, diagonal sprint (zigzag), exit to play, playing the ball, fast skip A through the training ladder), two repetitions of the track (20 s for the return to the start point), 2 min rest, two repetitions of the track (25 s for the return to the start point), 2 min rest, two repetitions of the track (30 s for the return to the start point), 2 min rest and two more repetitions of the track (30 s for the return to the start point);

(8th week) Seven exercises (slalom without ball, jumping on both legs, diagonal sprint (zigzag), exit to play, playing the ball, jump over the hurdles with one leg, fast skip A through the training ladder), two repetitions of the track (20 s for the return to the start point), 2 min rest, two repetitions of the track (25 s for the return to the start point), 2 min rest, two repetitions of the track (25 s for the return to the start point), 2 min rest, two repetitions of the track (30 s for the return to the start point), 2 min rest and two more repetitions of the track (30 s for the return to the start point).

The interval exercises (in the 3rd mesocycle) were performed on the 330 m-long circle track and the participants had to complete the lap in no more than 2 min. Moreover, in each week of the mesocycle the 30-, 40-, 50-, 60-m-long sprint was included in each lap, respectively. Additionally, inroads (30 m long, 20 s rest between each inroad) and ascents (15 m long, 10 s rest between each ascent) were performed (Figure 1). There was a 1 min rest between finishing inroads and starting ascents.

The 4th mesocycle consisted of continuous runs with load (with increasing distance and load, see legend for Figure 1) and interval exercises similar to those in 3rd mesocycle but with longer periods of sprint (Figure 1).

The training program was supervised by an experienced motor/personal trainer who observed and corrected the implementation of exercises, if needed.

### 2.3. Methods

As one of the study goals was to examine the effects of the proposed training program on the general health parameters, it was assumed to analyze as many parameters as possible. Such strategy would allow to detect any changes that may occur at different levels.

Participants’ body mass and body composition parameters (body mass index (BMI), basal metabolic rate (BMR), percentage of fat (FAT), fat free mass (FFM), total body water (TBW)), were determined using Body Composition Analyzer Tanita BC-418MA (Tanita, Tokyo, Japan).

During the experiment, blood samples were obtained five times from the median cubital vein: before the training program (baseline) and after each mesocycle (t1, t2, t3, t4) that is in the Monday morning following the last day (Saturday) of each mesocycle. These time points were chosen to examine organism’s response to the increasing physical loads realized during appropriate mesocycle. Each time, blood samples were taken into a 7.5 mL S-Monovette tube with ethylenediaminetetraacetic acid (EDTA K3, 1.6 mg EDTA/mL blood) (SARSTEDT AG & Co., Nümbrecht, Germany) and 7.5 mL S-Monovette tube for serum separation (SARSTEDT AG & Co., Nümbrecht, Germany). The blood was collected in laboratory conditions after an overnight fast, at 06:00, according to the standard diagnostic procedures. All analyses were performed immediately after the blood collection and/or serum separation. The biochemical tests were carried out using an Auto Chemistry Analyzer BM-100 (BioMaxima S.A., Lublin, Poland). Blood serum was used to determine metabolites (creatinine, lactate, urea, uric acid and bilirubin, both total and direct), albumin, total protein, ferritin, C reactive protein (CRP), lipid profile (triglyceride (TG), total cholesterol (TC), high-density, lipoprotein cholesterol (HDL-C), low-density lipoprotein (LDL-C) levels), enzyme activities (aminotransferases: aspartate (AST) and alanine (ALT), gamma-glutamyltransferase (GGT), alkaline phosphatase (ALP), creatine kinase (CK), lactate dehydrogenase (LDH), and amylase) and selected ions, namely iron, magnesium, phosphorus. All of the studied parameters were determined using a diagnostic method, following the manufacturer’s instructions (BioMaxima S.A., Lublin, Poland for most of them; QuimicaClinicaAplicada S.A., Amposta, Spain for ferritin; PZ Cormay S.A., Łomianki, Poland for lactate). All the analyses were verified using a multiparametric control serum, as well as a control serum of a normal level (BioNorm) and a high level (BioPath) (BioMaxima S.A., Lublin, Poland).

Complete blood count, including white blood cells (WBC), red blood cells (RBC), hemoglobin (HGB), hematocrit (HCT), mean corpuscular volume (MCV), mean corpuscular hemoglobin (MCH), mean corpuscular hemoglobin concentration (MCHC), and total platelets level (PLT) was obtained with a hematology analyzer ABX Micros 60 (Horiba ABX, Warsaw, Poland).

Motor performance was determined using four different tests to identify cardiorespiratory fitness (CRF), pace, and agility. CRF was determined by measuring time needed to cover the 1 km distance (outdoor run) and maximal multistage 20 m shuttle run test (Beep test; indoor run) [22,23] to estimate maximum oxygen uptake (VO_2_max). Pace was determined by measuring time needed to cover the 30 m distance in an indoor run. Agility was determined by using L test [24] in indoor conditions (athletics hall at a temperature of 20–23 °C). The times to overcome each section were measured using the Fusion Sport Smart Speed (Fusion Sport Pty Ltd., Brisbane, Australia).

### 2.4. Statistical Analyses

The statistical analyses were carried out using STATISTICA (data analysis software system), version 13 software (TIBCO Software Inc., Palo Alto, CA, USA, 2017), and the statistical significance was set at *p* < 0.05. To test the normal distribution within the subgroups, the Shapiro–Wilk test was used. Since none of the subgroups had a normal data distribution, nonparametric statistics were carried out. Results are presented as the medians and ranges (min–max). Significant differences between analyzed biochemical parameters obtained before and after the training program were calculated using the Mann–Whitney U test. Significant differences between analyzed time points (t0 vs. t1 vs. t2 vs. t3 vs. t4) were calculated using Friedman’s analysis of variance for repeated measures followed by a post-hoc Dunn’s test with Bonferroni correction.

## 3. Results

Motor performances of each individual participant were improved after the 4-month-long training program (Table 1). However, because of baseline heterogeneity in this group, and the fact that the sample size was quite small, the differences between baseline and post-training variables were not statistically significant. From this point of view, the personal change ratio (PC ratio) was calculated for the participants and presented in Table 1. The tests were performed only at the beginning and at the end of the training program because there was no rationale to perform strenuous tests more frequently, since the program was designed to increase participants’ motor performance during 4 months from one hand, and those tests may influence the biochemical and blood morphology results from the other.

All the participants at the beginning of the training program had a diet consultation and were instructed on proper nutritional practices. At each time point throughout the study, body composition was measured, but no significant differences were observed (Table 2).

Until the end of the second mesocycle no significant impact of training on serum enzyme activities was found (Table 3). However, significant differences were found in AST, LDH, ALT, and ALP activity after the third and/or fourth mesocycles (compared to baseline), respectively. The activities of aminotransferases, as well as LDH, were higher than baseline values while ALP activity was significantly lower than values found at the beginning of the training program. No significant changes were found in CK activity.

In contrast, during the training program, no significant changes were observed in most common clinical biomarkers, as well as in ions like iron, magnesium, phosphorus, or calcium (Table 4). However, the prolonged physical activity during these macrocycles caused a significant increase in the CRP levels (Table 4).

Even though no significant changes in RBC counts or HGB concentration were found, the width distribution of erythrocytes (RDW) and related parameters of MCH and MCHC were changing during the training program. Specifically, RDW values were significantly higher at the end (t4) of the macrocycles compared to baseline while MCHC was lower (Table 5). A significant decrease was also observed in PCT values. There were no statistically significant changes in any other blood morphology parameters (Table 5).

## 4. Discussion

The beneficial effects of physical activity are widely discussed in the literature [24,25,26,27,28,29,30]. Due to of the wide-ranging health benefits of PA, ensuring participant enjoyment through appropriate training interventions is important towards improving exercise compliance.

### 4.1. The Impact of the Training Program on Exercise Performance and Body Composition

The appeal of a training program may depend on the biomedical characteristics of the participants, as well as their exercise/sports history and economic situation. This study examined the impact of a macrocycle targeting improved exercise performance in a group of former athletes. Each individual participant noted small improvements in endurance, pace, and agility, while no significant improvement of body composition was observed. The most probable reason we did not observe changes in body composition is the participants may not have followed dietetic recommendations. It was summarized by Westerterp, who analyzed 23 exercise training research lasting from 2 up to 64 weeks, that although prescribed exercise caused energy imbalance, it was compensated by increased energy intake [31], that may confirm our observations regarding no changes in participants’ body composition.

### 4.2. The Impact of the Training Program on Biochemical Plasma Profile

The general biochemical profile of plasma enzyme activity indicated that during a training macrocycle, the enzymatic activity related to the motor apparatus, namely muscle cells, increases. In the case of both aminotransferases and LDH activities, the increase observed was related to the training program, since we could exclude any pathological cause. Generally, the increase in AST and ALT activity is related to liver or skeletal muscle injuries or heart attack [32], but there is increasing evidence that the increase in AST and ALT activity in professional athletes, specifically, is associated with the release of enzymes from muscle cells rather than liver pathology [8,33,34,35]. This study provides further evidence that among former athletes, interpretation of blood aminotransferases activities should consider the release of aminotransferases from skeletal muscle, especially given that no other significant changes in liver clinical parameters (e.g., GGT, bilirubin total and direct, total protein, albumin) during the 4-month-long study were observed. It is also worth noting that in comparison to our previous results [8,35,36], the current findings confirm that changes in AST and ALT activities emerge before any increase in CK activity. LDH activity is often considered a marker of muscle cell damage, as a rise in its concentration is concomitant with disruption to the sarcoma [37,38,39]. Further, evidence suggests the activity of total LDH and its isoenzymes is related to both endurance and strength of athletes [38,40,41]. The significant increase in LDH seen in the current study is in line with aminotransferases activity changes and the trend for an increase in CK levels.

The activity of blood ALP is a well-known diagnostic marker of mineralization and pathological disorders in bones [13,42,43]. The fluctuation of ALP activity found in this study is not consistent with the level of studied ions blood distribution (no statistical changes in ions concentration as a result of ALP activity). All studied ions and ALP activity were within population reference values for these analyses. However, there were no significant changes in calcium, phosphorus, or magnesium concentrations during the training program. Similarly, there were no changes in creatine, urea, uric acid, total protein, albumin, lipid profile (TC, LDL-C, HDL-C, TG), or iron metabolism (Fe, ferritin). The most probable explanation of these results is the fact that all the participants were healthy young men, without any disorders that could influence their biochemical profile. On the other hand, it can be assumed that the implemented training loads are properly prescribed and did not trigger abnormalities that profile.

Only C reactive protein was significantly increasing during the training program, and values found at the end of the macrocycle were higher than the reference values established for the general population. CRP blood concentrations are normally less than 2 mg/dL in healthy men [44]. Some data indicate that CRP concentrations are inversely related to physical fitness [45,46,47], but the impact of acute exercise may cause an increase [48,49,50] or no change [51] in CRP levels. There is also previous research indicating that long-term aerobic physical activity could decrease the CRP levels, which may be a relationship between CRP and blood lipids, body mass index (BMI), insulin metabolism, and/or blood pressure [46,51,52,53]. The significant increase in CRP level among the participants in the current study is consistent with the enzymatic profile described above and indicates that the training program caused muscular damage, therefore inducing pro- and anti-inflammatory responses. These responses provide further evidence that CRP is involved in the systematic responses to exercise-induced inflammation [54,55]. These processes stimulate muscular regeneration processes, so it can be considered as a positive effect of realized training program.

### 4.3. The Impact of the Training Program on Red and White Blood Cell Morphology

The tendency for participants to improve measures of exercise performance is reflected in their blood morphology. Though not all studied RBC-related parameters exhibited significant changes from pre- to post-training, maximal observed values did increase. For example, the changes observed in RDW, as well as other RBC-related parameters (e.g., MCH and MCHC) suggest that the erythropoiesis may increase during the last two mesocycles of a 4-month training cycle. The improvement of VO_2_max and the well-described correlation between O_2_ transport capacity and aerobic performance, as well as total hemoglobin and VO_2_max in athletes [56], helps to explain this observation.

WBC count is often reduced after a long-term training program [5,57]. There were no significant changes in WBC parameters in our study, but the highest values found among all participants progressively decreased at each time point. Taking the fact that WBC parameters (count and percentages of total WBC and their subpopulations) are the markers of general health status into account, the observed results of the present study may be considered as positive and confirming properly prescribed training loads and organism’s adaptation to them. Our previous works revealed post-effort increase in WBC parameter values caused not so much by the dehydration but related to immunological response to performed exercise [58,59,60].

It has recently been shown that the baseline mean platelet volume (MPV) may be a predictor of endurance performance [61,62]. However, other research indicates VO_2_max, time to fatigue, and running pace are not correlated with any platelet parameters [63]. Our study suggests that a training program focused on motor performance may cause a decrease in PCT that is related to a decrease in platelet counts (defined as a decrease in maximal values) from the beginning to the end of the training program. A common observation in professional athletes is increased fibrinolytic activity after exercise [64]. However, this acute response may be fully reversed. Signs of platelet “tiredness” (i.e., impaired functionality) have been observed in trained subjects during recovery [64,65]. Moreover, coagulation and fibrinolytic function parameters in athletes have also been found to be decreased or at least comparable to sedentary counterparts. It may be that such changes are favorable for physically active subjects, since they may be involved in protection against the risk of thrombosis and adverse cardiovascular events [64,65,66].

## 5. Conclusions

Implementation of an optimal short-term training plan can not only improve motor performance but also restore good health-related fitness habits. Each individual participant of the study noted small improvements in endurance, pace, and agility. We also confirmed that changes in AST and ALT activities emerge before any increase in CK activity that is important in monitoring of the training loads. Changes in red blood cell-related parameters suggest increase in erythropoiesis in the second half of the training cycle that allow to hypothesize e.g., the improvement of VO_2_max. However, this speculation requires further research.

## Figures and Tables

**Table 1:** Motor performance outcomes at baseline and post-training.

Variable	Participant No.	Baseline	Post-Training	PC Ratio
Time of outdoor run on 1 km distance (min)	1	3.57	3.32	0.93
	2	4.12	3.58	0.87
	3	4.32	3.51	0.81
	4	3.15	2.51	0.79
	5	3.38	3.05	0.90
	6	3.52	3.02	0.86
VO_2_ max (mL/kg/min)	1	43.06	48.86	1.13
	2	40.18	42.86	1.07
	3	36.86	44.33	1.20
	4	53.74	62.47	1.16
	5	47.34	57.07	1.20
	6	44.11	58.15	1.32
Pace (s)	1	4.23	4.06	0.96
	2	3.67	3.63	0.99
	3	4.21	4.01	0.95
	4	3.76	3.68	0.98
	5	4.02	3.89	0.97
	6	4.24	4.13	0.97
Agility (s)	1	17.86	16.82	0.94
	2	15.77	15.32	0.97
	3	16.68	15.47	0.93
	4	14.84	14.23	0.96
	5	14.85	14.52	0.98
	6	15.44	14.79	0.96

PC ratio—personal change ratio (post-training divided by baseline values).

**Table 2:** Participants’ body composition variables during the training macrocycle.

Variable	t0	t1	t2	t3	t4	*p*_ANOVA_
Weight (kg)	82.5	84.6	85.4	87.1	91.6	NS
	(75.6–98.5)	(77.0–96.6)	(75.3–98.1)	(79.2–97.5)	(81.9–96.3)	
BMI (kg/m^2^)	25.6	25.5	25.0	26.9	27.8	NS
	(23.0–28.8)	(23.4–28.9)	(23.2–28.7)	(24.4–28.5)	(25.3–28.4)	
BMR (kJ)	9150	9238	9199	8908	9253	NS
	(8602–10,309)	(8506–9560)	(8468–10,125)	(8782–878)	(9247–9782)	
Fat (%)	14.9	16.4	12.7	17.6	17.0	NS
	(7.0–21.5)	(8.7–22.7)	(7.1–18.5)	(9.8–18.5)	(15.9–20.9)	
Fat mass (kg)	12.2	13.6	11.1	15.3	15.8	NS
	(5.3–20.1)	(7.4–21.7)	(6.0–16.7)	(7.8–18.0)	(14.1–19.7)	
FFM (kg)	73.5	74	74.4	71.8	74.6	NS
	(69.8–102.0)	(69.1–77.2)	(69.1–81.4)	(71.4–79.5)	(74.5–78.8)	
TBW (kg)	53.8	54.2	54.5	52.6	54.6	NS
	(51.1–60.7)	(50.6–56.5)	(50.6–59.6)	(52.3–58.2)	(54.5–57.7)	

BMR—basal metabolic range; BMI—body mass index; Fat—the percentage of fat; FFM—fat-free mass; TBW—total body water. Data presented as medians (minimum−maximum) values. NS—not significant.

**Table 3:** Activities of participants’ selected plasma enzymes during the training macrocycle.

Variable	t0	t1	t2	t3	t4	*p*_ANOVA_	*p*_post-hoc_
Amylase (U/L)	53.2	62.8	56.8	96.1	63.8	NS	-
	(35.7–108.9)	(35.8–104.4)	(39.7–112.4)	(35.0–116.2)	(32.4–106.0)		
AST (U/L)	42.1	29.1	30.5	31.2	53.9	0.0242	0.0590 (t4 vs. t1)
	(36.1–58.9)	(25.5–37.4)	(25.7–53.8)	(26.1–67.5)	(34.0–73.3)		
ALT (U/L)	16.9	23.2	20.8	36.5	31.1	0.0156	0.0263 (t3 vs. t0)
	(14.7–26.8)	(15.7–25.6)	(17.7–75.4)	(22.3–75.3)	(25.7–35.4)		
CK (U/L)	275	131	191	124	280	NS	-
	(109–485)	(87.8–196.7)	(172–562)	(114–845)	(145–732)		
GGT (U/L)	16.0	20.2	17.1	16.5	18.2	NS	-
	(12.0–24.9)	(14.1–36.1)	(13.2–27.8)	(13.3–27.2)	(16.6–31.9)		
LDH (U/L)	280	314	356	290	383	0.0285	0.0399 (t4 vs. t0)
	(212–338)	(257–424)	(340–465)	(272–420)	(358–520)		
ALP (U/L)	112.3	88.2	81.6	61.2	78.0	0.0486	0.0348 (t3 vs. t0)
	(73.7–135.0)	(51.9–104.3)	(61.5–98.9)	(61–96.2)	(58.3–83)		

Data presented as median (minimum−maximum) values. AST—aspartate aminotransferase; ALT—alanine aminotransferase; CK—creatine kinase; GGT—γ-glutamyltransferase; LDH—lactate dehydrogenase; ALP—alkaline phosphatase. Significance levels of differences observed between analyzed time points (t0 vs. t1 vs. t2 vs. t3 vs. t4) were assessed using Friedman’s analysis of variance followed by post-hoc Dunn’s test with Bonferroni correction. NS—not significant.

**Table 4:** The values of biomarkers in participants’ blood during the training macrocycle.

Variable	t0	t1	t2	t3	t4	*p*_ANOVA_	*p*_post-hoc_
Cr (μmol/L)	101.1	110.0	111.0	110.0	105.5	NS	-
	(96.0–116.0)	(92.0–123.0)	(91.0–124.0)	(99.0–136.0)	(91.0–128.0)		
U (mmol/L)	8.4	6.7	7.3	7.2	6.4	NS	-
	(6.5–12.1)	(5.1–7.1)	(4.9–9.5)	(3.7–8.3)	(5.3–6.7)		
UA (μmol/L)	291	270	329	293	297	NS	-
	(191–402)	(247–386)	(214–406)	(249–306)	(269–351)		
DBIL (μmol/L)	6.02	5.54	5.43	5.37	6.85	NS	-
	(4.49–7.31)	(2.37–6.87)	(3.65–9.78)	(2.17–8.29)	(1.36–10.75)		
TBIL (μmol/L)	6.74	12.42	11.82	11.23	12.10	NS	-
	(4.05–9.98)	(7.87–14.2)	(8.42–15.25)	(8.10–13.36)	(4.98–16.82)		
total protein (g/L)	67.6	67.3	67.0	63.2	65.8	NS	-
	(63.4–76.2)	(64.6–74.8)	(63.7–71.0)	(61.1–69.1)	(60.3–70.1)		
albumin (g/L)	49.6	48.2	47.4	46.7	46.3	NS	-
	(44,4–50.7)	(45.2–50.6)	(46.9–49.2)	(43.7–47.5)	(44.5–49.2)		
CRP (mg/L)	0.0	0.6	2.3	8.05	8.13	0.005	0.0387 (t3 vs. t0) 0.0126 (t4 vs. t0)
	(0.0)	(0.0–1.6)	(0.0–3.3)	(0.84–18.9)	(1.77–14.12)		
TC (mmol/L)	3.82	4.39	4.09	4.50	4.01	NS	-
	(3.27–4.45)	(3.54–4.86)	(3.46–4.74)	(3.29–4.64)	(3.55–4.74)		
LDL-C (mmol/L)	2.02	2.24	2.01	2.40	2.13	NS	-
	(1.11–2.60)	(1.14–2.61)	(1.43–2.61)	(1.12–2.55)	(1.05–2.44)		
HDL-C (mmol/L)	1.40	1.80	1.66	1.84	1.75	NS	-
	(1.15–1.84)	(1.33–2.13)	(1.45–1.76)	(1.19–1.92)	(1.42–2.22)		
TG (mmol/L)	0.83	0.93	0.97	0.59	0.90	NS	-
	(0.50–1.53)	(0.43–1.77)	(0.50–1.51)	(0.54–2.00)	(0.60–1.04)		
ferritin (ng/mL)	144.5	143.2	141.7	144.7	143.0	NS	-
	(140.9–146.2)	(141.6–147.1)	(139.9–146.3)	(143.4–147.7)	(140–147)		
Fe (μmol/L)	19.7	21.1	19.7	18.9	15.6	NS	-
	(12.7–31.1)	(18.8–28.2)	(17.9–24.2)	(18.5–20.8)	(11.6–31.5)		
Mg (mmol/L)	0.85	0.8	0.79	0.86	0.84	NS	-
	(0.81–0.91)	(0.74–0.89)	(0.78–0.82)	(0.83–0.87)	(0.78–0.87)		
P (mmol/L)	1.53	1.36	1.21	1.19	1.26	NS	-
	(1.36–1.55)	(0.81–1.64)	(1.05–1.41)	(0.00–1.42)	(0.87–1.52)		
Ca (mmol/L)	2.58		2.53	2.56	2.40	NS	-
	(2.52–2.74)		(2.44–2.54)	(2.42–2.62)	(2.37–2.48)		

Data presented as median (minimum−maximum) values. Cr—creatinine; U—urea; UA—uric acid; DBIL—direct bilirubin; TBIL—total bilirubin; CRP—C-reactive protein; TC—total cholesterol; LDL-C—low-density lipoprotein cholesterol; HDL-C—high-density-lipoprotein cholesterol; TG—triglycerides; Fe—iron; Mg—magnesium; Ca—calcium; P—phosphorus. Significance levels of differences observed between analyzed time points (t0 vs. t1 vs. t2 vs. t3 vs. t4) were assessed using Friedman’s analysis of variance followed by post-hoc Dunn’s test with Bonferroni correction. NS—not significant.

**Table 5:** Participants’ blood morphology during the training macrocycle.

Variable	t0	t1	t2	t3	t4	*p*_ANOVA_	*p*_post-hoc_
RBC (10^9^/L)	4.92	4.87	5.08	4.84	5.12	NS	-
	(4.13–5.07)	(4.36–5.22)	(4.98–5.17)	(4.57–5.17)	(4.48–5.93)		
HGB (mmol/L)	8.60	8.40	8.45	8.60	8.85	NS	-
	(7.40–9.40)	(7.60–9.00)	(8.40–8.50)	(7.7–9.1)	(7.50–10.3)		
HTC (L/L)	0.46	0.46	0.47	0.47	0.49	NS	-
	(0.39–048)	(0.41–0.49)	(0.46–0.48)	(0.41–0.49)	(0.42–0.57)		
MCV (fL)	95	95	93	95	96	NS	-
	(92–95)	(92–96)	(90–96)	(91–97)	(93–97)		
MCH (fmol)	1.80	1.75	1.66	1.76	1.72	0.0176	0.013 (t2 vs. t0)
	(1.74–1.85)	(1.68–1.76)	(1.62–1.70)	(1.68–1.78)	(1.67–1.77)		
MCHC (mol/L)	19.1	18.4	17.9	18.5	18.0	0.0018	0.0098 (t2 vs. t0) 0.0056 (t4 vs. t0)
	(18.7–19.4)	(13.3–18.5)	(17.8–18.1)	(18.3–18.6)	(17.9–18.3)		
RDW (%)	10.8	11.4	11.4	11.3	11.6	0.0332	0.0178 (t4 vs. t0)
	(10.6–11.3)	(11.0–11.9)	(10.9–1.9)	(11.3–11.6)	(11.4–12.0)		
WBC (10^9^/L)	8.1	7.1	6.8	7.4	6.1	NS	-
	(6.0–10.2)	(6.3–9.6)	(6.1–7.4)	(5.1–7.8)	(4.2–6.8)		
LYM (%)	39.0	38.4	45.0	42.9	38.8	NS	-
	(27.0–49.0)	(19.6–47.1)	(42.1–47.9)	(39.0–45.4)	(18.7–41.5)		
MON (%)	3.8	4.5	4.4	4.5	4.4	NS	-
	(3.1–5.5)	(4.2–5.4)	(4.0–4.8)	(4.2–4.6)	(2.7–5.9)		
GRA (%)	56.9	56.7	50.6	52.5	56.8	NS	-
	(47.3–70.4)	(48.4–76.2)	(48.1–53.1)	(50.4–56.5)	(52.6–78.6)		
LYM (10^9^/L)	2.90	3.10	3.00	3.00	2.05	NS	-
	(2.10–4.00)	(1.30–3.60)	(2.9–3.1)	(2.3–3.1)	(1.00–2.70)		
MON (10^9^/L)	0.20	0.30	0.25	0.30	0.20	NS	-
	(0.20–0.50)	(0.20–0.40)	(0.20–0.30)	(0.20–0.30)	(0.10–0.30)		
GRA (10^9^/L)	5.30	3.80	3.50	4.00	3.8	NS	-
	(3.50–5.80)	(3.30–5.60)	(3.00–4.00)	(2.6–4.5)	(2.4–4.6)		
PLT (10^9^/L)	236	259	290	254	234	NS	-
	(127–326)	(133–280)	(282–297)	(236–306)	(181–250)		
MPV (fL)	6.9	7.1	7.6	7.5	7.1	NS	-
	(6.8–9.5)	(6.5–9.6)	(7.3–7.8)	(6.8–8.1)	(6.5–7.5)		
PCT (10^−2^L/L)	0.18	0.18	0.22	0.19	0.17	0.0426	0.049 (t4 vs. t2)
	(0.12–0.22)	(0.13–0.21)	(0.21–0.23)	(0.17–0.23)	(0.12–0.18)		
PDW (%)	14.7	14.9	16.1	16.1	14.7	NS	-
	(14.6–17.9)	(14.0–20.1)	(15.8–16.3)	(15.8–16.5)	(14.2–15.9)		

Data presented as median (minimum−maximum) values. RBC—red blood cells count; HGB—hemoglobin; HTC—hematocrit; MCV—mean corpuscular volume; MCH—mean corpuscular hemoglobin; MCHC—mean corpuscular hemoglobin concentration; RDW—red blood cell distribution width; WBC—white blood cells count; LYM—lymphocytes; MON—monocytes; GRA—granulocytes; PLT—platelets count; MPV—mean platelet volume; PCT—plateletcrit; PDW—platelet distribution width. Significance levels of differences observed between analyzed time points (t0 vs. t1 vs. t2 vs. t3 vs. t4) were assessed using Friedman’s analysis of variance followed by post-hoc Dunn’s test with Bonferroni correction. NS—not significant.
